# Round the Hospitals

**Published:** 1919-10-04

**Authors:** 


### ROUND THE HOSPITALS.

The Duke of Connaught visited on Saturday September 27 the Royal Albert Orphanage at Camberley, on the occasion of a presentation to the superintendent and matron, Mr. and Mrs. W. J. Burn, upon their retirement after sixteen years' service in the institution. Mr. and Mrs. Burn received from the hands of the Duke on behalf of the Lady Patricia Ramsay, the President, and the members of the Ladies Committee, a silver salver, together with many graceful acknowledgments of their united work in the orphanage.

It is announced by the Ministry of Health that in accordance with the provisions of the Ministry of Health Bill it will seek advice on a variety of subjects within its province from advisory bodies composed of men and women having full practical knowledge of the questions on which they are asked to advise. These advisory bodies will consist of twenty members each, of whom at least half will be women in the case of the Council on General Health Questions, and they may for special purposes appoint committees on which other persons may be co-opted. So

**ROUND THE HOSPITALS**—(*continued*).

that particular problems will be considered with the help of those who have made them a particular study. In the case of England there will be four Consultative Councils on (1) Medical and Allied Services, (2) National Health Insurance, (3) Local Health Administration, (4) General Health Questions.

---

To the Council of the Medical and Allied Services will be referred such problems as the extension and development of medical, nursing, and midwifery work. The members have been chosen from the fields of specialist, medicine and surgery, general practice, both private and insurance, the public health services, the work of women in medicine (particularly the care of mothers and infants), hospital administration, and the application of other branches of science to medicine. The Committee will include the following:—Sir Bertrand Dawson, Chairman; Col. C. J. Bond, F.R.C.S., Vice-Chairman (Leicester); Mr. Norman Bennett, L.D.S. Eng. (Brit. Dent. Asso.); Professor R. A. Bolam, M.D. (Newcastle); Dr. Victor Bonney, F.R.C.S. (Chelsea Hospital for Women); Professor T. E. Hill, M.B. (Durham); Professor Gowland Hopkins (Cambridge) (Off. Analyst to the Home Office); Miss M. H. F. Ivens, M.B., M.S. (Liverpool); Dr. Janet E. Lane-Claypon (King's College for Women, London); Mr. A. Linnell, M.R.C.S. (Northampton); Mr. H. G. Dain, M.B., M.R.C.S. (Birmingham); Mr. A. Fulton, M.B. (Nottingham); Sir William Glyn-Jones (Sec. Pharm. Society); Dr. T. A. Goodfellow (Manchester); Dr. G. E. Haslip (Treasurer, Brit. Med. Asso.); Dr. J. A. Macdonald (Taunton); Mr. W. E. Morris (Sec., London Hospital); Dr. John Robertson (Birmingham); Dr. T. W. Shore (St. Bartholomew's Hospital); Professor Sir William Tilden, F.R.S.

---

Universal satisfaction is felt by all who have set their hands to the alleviation of suffering at the news that an agreement has been signed by the British Red Cross Society and the Order of St. John of Jerusalem, under which the harmonious working of the twin Societies in war will be carried forward into the new tasks of peace. A Joint Council has been set up, consisting of an equal number of members of each body. It is charged with the control of the work, and alone has power to authorise appeals for funds. Members of the existing Joint War Committee will be members of the new Joint Council. The Hon. Sir Arthur Stanley, Chairman of the Joint War Committee, states that no actual beginning can be made until the Privy Council has extended the existing charter so that it may be brought into line with Article XXV. of the Covenant of the League of Nations. The delay in granting this charter, which no one anticipates will be refused, is responsible for the delay in getting to work, which everyone connected with the Red Cross and the Order of St. John finds very irksome.

Great interest attaches to the list of urgent tasks which Sir Arthur Stanley outlined as shortly to be brought before the Joint Council. They include the care of sick and wounded sailors and soldiers, whether demobilised or not; the care of all suffering from tuberculosis, and particularly of sailors and soldiers; child welfare; work parties to provide garments and necessaries for hospitals and health institutions; voluntary aid in all branches of nursing, health, and welfare work, ancillary to the Ministry of Health; Red Cross war and peace hospital library; home service and ambulance work. Sir Arthur Stanley foreshadows a comprehensive campaign against tuberculosis throughout the entire country, carried out in concert with the National Association for the Prevention and Cure of Tuberculosis, of which he has just accepted the chairmanship. The old V.A.D. organisations are to be revised, and must be approved by the Army Council before a new start is made. The general principle of a central committee in each county, with a county director, is to be maintained. As Sir Arthur justly observes, "It has been found to answer well, and as it has stood the strain of war, it may be hoped that it will serve equally well in time of peace."

---

A festival dinner takes place on Monday, November 3, at the Great Eastern Hotal, on the occasion of a gathering of the East End Traders' Association, at which Mr. Walter Preston will preside. Always having in mind the great hospital which ministers to the needs of the East End population, the proceeds of the festival are to go to the London Hospital.

---

The Central Midwives Board, having now come under the jurisdiction of the Ministry of Health, issues a statement with regard to the proposed re-constitution of the Board. At the beginning of this year, the Board resolved that it was expedient to modify its constitution so that it should consist of the following members: (*a*) three persons appointed by the Lord President of the Council (Minister of Health), of whom one shall be a certified midwife on the English Roll, preferably one who is or has been an inspector of midwives appointed by a Local Supervising Authority. (*b*) Four registered medical practitioners appointed as provided by the Midwives Act 1902; (*c*) one certified midwife appointed by the Incorporated Midwives Institute; (*d*) One person appointed by the Q.V.J.I.N.; (*e*) One person appointed by the Association of County Councils; (*f*) One by the Association of Municipal Corporations; (*g*) One by the Society of Medical Officers of Health. It is proposed that the representation of the Royal British Nurses Association shall cease. The effect is to increase the members of the Board from nine to twelve, a real advantage considering the amount of work to be done. The Board contains no certified midwife representing midwives in general, and as there is a strong feeling that midwives should be represented on the governing body of their profession it is suggested

**ROUND THE HOSPITALS—*(continued)*.**

that two midwives should be included in the constitution of the Board. There can hardly be two opinions on the expediency of the alterations as proposed. The representation of all bodies directly engaged on the work it has to transact will, it is believed, appreciably strengthen its hands by the contribution of added knowledge and experience to its deliberations.

---

The Tynemouth Guardians have been very well advised in the steps they are taking to secure a regular rotation in the supply of probationer nurses. In view of the fact that eight of the present staff will shortly complete their training, and that on their departure the numbers of the untrained and partly-trained probationers will be out of the usual proportion, they have decided to offer seven of the nurses an adequate inducement to remain an extra year in their service. It is proposed to appoint them temporary staff nurses when they have obtained their certificates, at a salary of £25 plus war bonus, and to give them opportunities for taking such midwifery training as shall qualify them for the C.M.B. examination. In order to carry this out, the medical officer of the institution has applied for recognition as a lecturer in midwifery, and an outside midwife is to give practical training at a fee of £5 for each nurse trained.

---

It is with pleasure we are able to announce that following a meeting at Newcastle the National Poor-Law Officers Association adopted a programme which considerably revises the existing working conditions. In future there is to be a forty-four hour week with one whole day off in seven. The salaries are to be not less than the present day rates *plus* the Civil Service scale of war bonus, and overtime pay. For all officers at least three weeks' annual leave, and for nurses a minimum of four weeks. Is this generous scheme to become operable in all Poor-Law institutions? If so, there are indeed better times on the horizon for nurses, especially within infirmaries.

---

An interesting ceremony takes place at the Great Northern Central Hospital on Thursday, October 9, at 3 o'clock, when Lady Islington will distribute badges and bars to members of the League of Roses. The Chairman of the League (Miss F. M. Roby) will preside, and the event is one which is of great interest to all those who have had past connections with the hospital.

---

The annual reunion of the Crumpsall Infirmary nurses was suspended during the war. This famous training centre has had a difficult year, the work having been exceptionally heavy during 1918. Repeated outbreaks of influenza and pneumonia, with a high mortality, have taxed the medical wards severely. That the training-school has had its own trials is evidenced by the analysis of probationers supplied for March 31, 1919. Out of a total of 106 only eleven were in their third year of training; thirty-two were in their second year; and sixty-three in their first. Altogether forty-two nurses were examined and certificated, and two failed. There were three passes for the C.M.B. examination, and one failure. The nurses receive training in children's diseases at the Booth Hall Infirmary, and a special department for babies under eighteen months, with an average of fifty occupied cots, affords valuable material for special training in the Infirmary itself. The obstetrical and gynæcological work comprised 243 cases, with 352 cases in the maternity department. There is also a large department for $x$-ray, electrical, and massage treatment, and extensive additions have been made to the department for venereal diseases.

---

The recent discovery that fatigue is located in the cells of the nervous system and not in the muscles sheds a ray of light on the causes which induce exhaustion in a nurse after a long day spent in a patient's room without any very arduous duties to perform. The upholders of the traditional day or night of ten or twelve hours on duty are apt to emphasise the sedentary character of the work and the absence of muscular or mental effort required. They forget that the very monotony and confinement tax the nerves to the utmost extent. After such a spell of duty indoors the truest relaxation for the nerves lies in the open air, but if possible it should be taken in some more exhilarating form than that of an aimless wandering through streets, during which the same old current of ideas persists. When the body has been refreshed by air and movement the mind is ready for fresh material, and the stimulus supplied to it in the lecture-room banishes the feeling of fatigue by calling on fresh and dormant powers. Far too much stress is commonly laid on sensations of fatigue. They vanish automatically as soon as they can be driven out by a new interest, but without this they will persist even while the body is in complete repose.

---

The International Conference of Women Doctors, lately sitting in New York, found a congenial topic in a discussion on clothes, for however learned a woman may be her primary instinct to criticise the garb affected by other women is never entirely lost. Some hard things, by no means too hard, however were said on the type of garments which Mr. Wells describes as "sexual trappings," and we learn without astonishment that "the American wife is nothing short of a shop-window for her husband," her clothes indicating her husband's financial standing. In this country a healthy reaction against the absurd decrees of the man milliner has made great strides during the war, many women of ample means having tacitly adopted resistance to whatever curtails their movements or offends good taste. In this respect professional and business women alike set a good example, and put the frivolous to shame by demonstrating the beauty of garments perfectly adapted to the purpose in hand.

---

The Railway Strike is the universal subject of conversation. Food and Transport are the

**ROUND THE HOSPITALS**—(*continued*).

problems most prominently occupying the minds of people all over the country. Hyde Park has again established itself as the hub of London, for from the neighbourhood of the Achilles Statue the feeding of the Metropolis is to be maintained. The People's Park is therefore temporarily closed to the public, and from within can be seen the machinery of food distribution at work. The regulations previously in force regarding rationed food will automatically come into operation both as regards the use of ration books or cards by proprietors of residential establishments, and by institutions in the same way for their officers, and as regards the quantities consumed by their inmates. To prevent unnecessary suffering it is understood that hospitals, asylums, and workhouses, etc., will receive primary consideration.

The railway stoppage became complete very early on Saturday at Derby, which as the headquarters of the Midland system is one of the most important railway centres in the provinces. Only three trains ran on Saturday, one bound for the North included among its passengers 200 Red Cross Nurses anxious to reach Scotland, but who probably got no further than York.

The maternity home at Plymouth for sailors' wives, with hostel attached where children can be well-cared for while their mothers are being confined, has proved almost embarrassingly popular, and the demand for an extension is widespread in the wide district it serves. An urgent appeal for £50,000 has been issued by Lady Beatty, Lady Maud Warrender, and Mrs Alice Mildmay to provide the funds needed to enlarge the existing maternity home and the hostel for district nurses attached to it, as well as to assist similar institutions at other naval bases. The Navy League has promised £30,000, to be divided in equal proportions between Plymouth, Portsmouth, and Chatham. It is well said that the Empire can acknowledge its debt to the sailors in no better way than by providing medical and nursing attention, and every comfort for their wives at the most critical moments of their lives. Subscriptions, small or large, will be gratefully received by Sir James Gilder, K.C.V.O., 23 Queen Anne's Gate, S.W. 1, and it is noted that "every shilling helps."

A correspondent, "M. L. N.," to whom we would express our best thanks, sends us a copy of a novel which she suggests we do not mention lest it increase the sale of a very odious production. She justly considers it presents a picture of nurses in training calculated to do great harm to women who may be contemplating getting trained as nurses, and indeed to all who have no knowledge of hospital life as it really is. We should judge it to be written by a woman who has had such a glimpse of the profession as may be gained by a candidate dismissed after her trial month. Its tone is unspeakably vulgar. The heroine in training has been co-respondent in a divorce case, and her bosom friend in the training-school is the daughter of a bankrupt earl who has committed suicide. Needless to say, one of the specialists "features" as the hero. Every gruesome detail of the operating-theatre is pressed into service, and the language would be considered disagreeable in any respectable servants' hall. Messrs. Mills & Boon have not enhanced their reputation by the publication, in a cheap edition, of so ignorantly written and mischievous a story.

A few months ago the Willesden Urban District Council proposed a scheme by which the municipal hospital might be used as a training centre for midwives, but now the requisite sanction cannot be obtained. At a meeting of the Health Committee consideration was given to the correspondence which has taken place between the Medical Officer of Health and the Central Midwives' Board, and the Committee now reports that it is not able to follow the Board's decision refusing the application and is communicating with the Ministry of Health on the subject. It is interesting to note that the Willesden Council is showing itself disposed to co-operate with philanthropical societies for the rescue of the unmarried mother. The Hampstead Ruri-decanal Association recently applied to the Council to aid them by a grant of £10 in sending an unmarried mother and child to a home. The Council has agreed, subject to the Medical Officer of Health being satisfied that the woman was a resident of Willesden and to an application being made to the Ministry of Health for a grant in respect of this expenditure. This, so far as we know, is the first grant of the kind to be made by any local authority.

Nurse Muriel Dorothy Northen is another brave volunteer. She joined a Voluntary Aid Detachment as soon as war broke out, served as a V.A.D. nurse in Cambridge Hospital, and then volunteered for overseas. On her way to Salonika in 1916 her ship was torpedoed, and for services rendered on that occasion she was made an Associate of the Royal Red Cross. The following year when on her way to Egypt she had a second experience of the horrors of the torpedo and was mentioned in dispatches for her brave conduct. Her home is in Lewisham, and the Deptford Borough Council have resolved that her name be recorded on the Honours Board in the Council Chamber, that an illuminated record be presented to her commemorating her devotion to duty, and that she be invited to sign the Borough Roll of Honour.

Some of our readers may have noticed that active preparations are being made for the erection of the statue to Miss Cavell on its chosen site opposite St. Martin's Church, Trafalgar Square. The concrete foundations are now being laid. The statue will be a full-length figure of Nurse Cavell facing south. The entire memorial will be 30 feet high.

